# SMARCA4 deficiency in small cell lung cancer: A case report and narrative review of the literature

**DOI:** 10.17305/bb.2024.11154

**Published:** 2025-01-27

**Authors:** Andreas M Matthaiou, Ioannis Tomos, Nikoleta Bizymi, Ioannis Vamvakaris, Nektarios Anagnostopoulos, Aikaterini Papadopoulou, Kalliopi Angelou, Grigoris Stratakos, Adamantia Liapikou

**Affiliations:** 1 5th Department of Respiratory Medicine, Sotiria Thoracic Diseases General Hospital of Athens, Athens, Greece; 2 Laboratory of Molecular and Cellular Pneumonology, Medical School, University of Crete, Heraklion, Greece; 3 Respiratory Physiology Laboratory, Medical School, University of Cyprus, Nicosia, Cyprus; 4 Laboratory of Pathology, Sotiria Thoracic Diseases General Hospital of Athens, Athens, Greece; 5 1st Respiratory Medicine Department of the National and Kapodistrian University of Athens, Sotiria Thoracic Diseases General Hospital of Athens, Athens, Greece; 6 New Intensive Care Unit, Sotiria Thoracic Diseases General Hospital of Athens, Athens, Greece

**Keywords:** SWItch/sucrose non-fermentable, SWI/SNF, SWI/SNF related BAF chromatin remodeling complex subunit ATPase 2, SMARCA2, SWI/SNF-related BAF chromatin remodeling complex subunit ATPase 4, SMARCA4, small cell lung cancer, SCLC

## Abstract

SWItch/sucrose non-fermentable (SWI/SNF) is a large protein complex with a central role in chromatin remodeling and genome transcription. The catalytic subunits of the SWI/SNF related BAF chromatin remodeling complex subunit ATPase 2 (SWI/SNF SMARCA2; also called BRM) and SWI/SNF related BAF chromatin remodeling complex subunit ATPase 4 (SMARCA4; also called BRG1) are encoded by the *SMARCA2* and *SMARCA4* genes, respectively, and are mutually exclusive. Loss of either SMARCA2 and/or SMARCA4 has been previously reported in several types of malignant solid tumors of the gastrointestinal and genitourinary tract. So far, their absence in non-small cell lung cancer (SCLC) has been observed in a series of studies involving primary tumors and cell lines, where it is associated with loss of differentiation and heightened tumorigenic potential leading to an unfavorable prognosis. SMARCA2 and SMARCA4 deficiency is frequent in solid predominant adenocarcinomas and tumors with low levels of bronchial epithelial markers, including thyroid transcription factor 1. A rare case of SMARCA4 deficiency in SCLC is described. A 57-year-old male patient, with no medical history of past illness, was admitted to our center for the investigation and management of a space-occupying lesion in the right upper lung lobe with tracheal and mediastinal infiltration. Biopsy of a lymph node in the right supraclavicular region was diagnostic for SCLC with regional loss of SMARCA4. The patient demonstrated progressive respiratory failure and clinical deterioration and eventually deceased despite intubation and transfer to the intensive care unit. This case indicates that SMARCA4-deficient SCLC may present with an aggressively deteriorating phenotype with poor outcomes for the patients.

## Introduction

SWItch/sucrose non-fermentable (SWI/SNF) is a large protein complex that plays a central role in chromatin remodeling and genome transcription. It regulates crucial cellular processes, including cell proliferation, cell differentiation, and DNA repair. SMARCA2 (also called BRM) and SMARCA4 (also called BRG1) are two mutually exclusive catalytic subunits of SWI/SNF and are encoded by the *SMARCA2* and *SMARCA4* genes, respectively. Their function as adenosine triphosphatases provides the energy required for altering chromatin organization and regulating transcription [1].

Loss of either SMARCA2 or SMARCA4, or both, has been observed in several types of malignant solid tumors of the gastrointestinal and genitourinary tracts. It has been associated with a highly undifferentiated phenotype and an unfavorable clinical course. Small cell carcinoma of the ovary, hypercalcemic type—a rare malignancy of the female reproductive system—currently represents the prototype of SMARCA4-deficient neoplasms, as SMARCA4 loss is found in all cases. In endometrial carcinomas, SMARCA4 deficiency is associated with a dedifferentiated or anaplastic phenotype. These neoplasms are either entirely undifferentiated or composed of both differentiated and dedifferentiated parts, suggesting that SWI/SNF dysfunction occurs as a secondary genetic alteration, leading to the morphological shift of the tumors [2, 3].

Loss of SMARCA2 and SMARCA4 is occasionally observed in non-small cell lung cancer (NSCLC) and is associated with poor clinical outcomes. Here, we describe a rare case of small cell lung cancer (SCLC) with SMARCA4 loss, characterized by an aggressive phenotype, and further discuss the molecular, pathological, and clinical features of SMARCA2 and SMARCA4 deficiency in lung cancer.

## Case report

**Figure 1.**
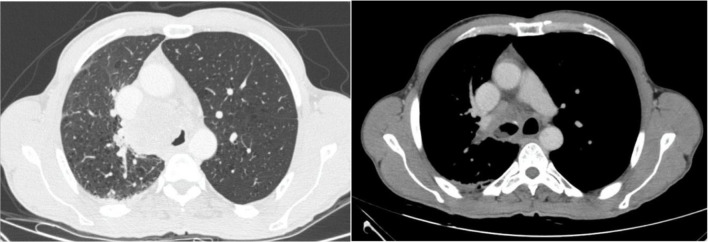
Chest computed tomography scans showing a large mass in the right upper lung lobe with tracheal and mediastinal infiltration.

A 57-year-old male, current smoker (100 pack-years), with no medical history of past illness, was admitted to the Sotiria Thoracic Diseases General Hospital of Athens due to exertional dyspnea and stridor over the past week. Chest computed tomography revealed a large mass in the right upper lung lobe with tracheal and mediastinal infiltration (Figure 1). Bronchoscopy was performed and revealed an exophytic lesion invading the lower part of the trachea, causing significant narrowing of its lumen, as well as infiltration and complete obstruction of the right main bronchus. Debulking of the tracheal tumor and restoration of patency in the right main bronchus were successfully performed using rigid bronchoscopy.

**Figure 2.**
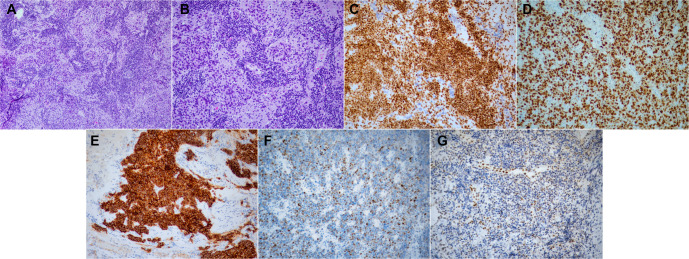
**Histopathological examination of lymph node biopsy (magnification ×10), which revealed infiltration by small cell lung cancer of low differentiation with regional loss of SMARCA4, complete loss of CK18 and RB1, and expression of Ki-67 in more than 90% of the cells.** (A and B) Hematoxylin–eosin stain: (A) 10× magnification; (B) 20× magnification; (C–G) Immunohistochemistry stains: (C) SMARCA4 stain; (D) Ki-67 stain; (E) CD56 stain; (F) CK18 stain; (G) RB1 stain. SMARCA4 and RB1 stains were performed twice for the confirmation of the results. CK18: Cytokeratin 18; RB1: Retinoblastoma 1.

Biopsy of the tumor initially revealed poorly differentiated malignant cells surrounded by large amounts of necrotic debris. Excision of a lymph node in the right supraclavicular region, which displayed clinically malignant features, was subsequently performed, and biopsy confirmed a diagnosis of SCLC. Cellular morphology was consistent with SCLC, characterized by small-sized, round-shaped cells with scant cytoplasm, fine granular chromatin, and inconspicuous nucleoli, which excluded a mesothelial origin of the lesion. Immunohistochemical analysis was positive for synaptophysin, chromogranin, and insulinoma-associated protein 1. The neoplasm demonstrated regional loss of SMARCA4 in areas where neuroendocrine markers were positive. Furthermore, immunonegativity for other markers, including CD3, CD20, CD30, CD56, cytokeratin (CK) 18, low-molecular-weight keratin, leukocyte common antigen, p40, placental alkaline phosphatase, retinoblastoma 1, and thyroid transcription factor 1 (TTF-1), was observed. A high mitotic index was evident, with Ki-67 expression in more than 90% of the cells (Figure 2).

Further staging of the disease and investigation of metastatic lesions in visceral organs and bones were not conducted due to the clinical deterioration of the patient. He experienced progressive respiratory failure, was intubated, and transferred to the intensive care unit, where he underwent serial rigid bronchoscopy sessions for debulking of the endotracheal lesion. Ultimately, he succumbed to respiratory failure after a prolonged hospital stay.

### Ethical statement

Written informed consent has been obtained from the patient to publish this paper. The principles outlined in the Declaration of Helsinki were followed.

## Discussion

### Loss of SMARCA2 and SMARCA4 in lung cancer

Reisman et al. [4] were the first to describe the loss of SMARCA2 and SMARCA4 in NSCLC two decades ago. They used Western blotting to demonstrate the molecular phenomenon in 30% of NSCLC cell lines and immunohistochemistry to show concomitant loss of SMARCA2 and SMARCA4 in 10% of primary NSCLC tumors across 41 adenocarcinoma and 19 squamous cell carcinoma (SCC) cases. Additionally, patients with SMARCA2 and SMARCA4 deficiency had a statistically significant decrease in survival. Since then, several studies involving primary tumors, cell lines, and animal models of lung cancer have shed more light on the role of SMARCA2 and SMARCA4 deficiency in the disease. Only one study, by Li et al. [5], associated the upregulated expression, rather than the deficiency, of SMARCA4 with higher malignancy grades. This study further suggested that SMARCA4 might play an important role in hypoxia-induced transcription of genes involved in cancer cell proliferation and migration.

Screening of the entire coding sequence of *SMARCA4* in 59 lung cancer cell lines revealed mutations in 24% of the cell lines, which were much more common in NSCLC (35%) than in SCLC (5%) [6]. Immunohistochemical analysis of 93 primary lung adenocarcinomas revealed loss of SMARCA2 and SMARCA4 in 17% and 12% of cases, respectively. The authors concluded that the loss of either or both SMARCA2 and SMARCA4 led to the progression of lung adenocarcinoma into a solid-predominant tumor and to epithelial–mesenchymal transition, with loss of the bronchial epithelial phenotype [7]. SMARCA4-deficient NSCLC was correlated with male sex, older age, smoking history, larger tumor size, and a higher tumor proliferation index (higher Ki-67), among other clinicopathological features, while patients demonstrated worse prognosis compared to SMARCA4-intact cases [8].

Positive nuclear staining of SMARCA2 and concomitant nuclear staining of SMARCA2 and SMARCA4 were both associated with better five-year survival in 300 NSCLC cases (150 adenocarcinoma and 150 SCC). However, positive membranous (i.e., extranuclear) staining of SMARCA2 was associated with worse five-year survival. These findings indicated that SMARCA2 and SMARCA4 participate in two distinct, functionally complementary chromatin-remodeling complexes and that their expression and cellular localization could serve as useful markers for NSCLC prognosis [9].

In the study by Herpel et al. [10] involving 316 specimens of NSCLC, loss of SMARCA4 was shown in 5.1% of cases, loss of SMARCA2 in 4.8%, and concurrent loss of both markers in only four cases. Importantly, most SMARCA4-deficient tumors demonstrated a clearly differentiated phenotype, either adenocarcinoma or SCC, suggesting that loss of SMARCA4 in NSCLC is a primary genetic driver event, in contrast to endometrial carcinomas, where it results from a second or third hit and leads to dedifferentiation.

Experiments in both NSCLC cell lines and primary tumors revealed that the loss of SMARCA4 led to disturbed cellular morphology and heightened tumorigenic potential. Alterations in chromatin organization included nucleosome repositioning toward transcriptional initiation sites of cancer-associated genes [11]. Significantly lower levels of SMARCA4 were found in lung cancer cell lines compared to normal lung tissues. Restoration of SMARCA4 was associated with decreased lung cancer cell proliferation, metastasis, and epithelial–mesenchymal transition, an effect attributed to its positive regulatory role in the expression of miR-148b, which inhibits the WNT1/β-catenin signaling pathway [12].

Multi-omics analysis and in vitro experiments identified *SMARCA2* as a tumor suppressor gene with decreased expression in lung cancer, showing that its inactivation can be epigenetically driven by promoter hypermethylation [13]. Two polymorphic insertion promoter sequence variants in *SMARCA2*, prevalent in 20% of Caucasians, were strongly correlated with the loss of SMARCA2 in both NSCLC cell lines and primary NSCLC cells. These variants were further characterized as markers of higher risk for lung cancer development among smokers [14]. The two *cf* promoter sequence variants and their resultant decreased gene expression were associated with worse overall survival [15].

Knockout experiments of *SMARCA4* in murine lungs exposed to a carcinogen showed that loss of SMARCA4 was associated with an increased number and size of lung tumors and higher levels of cell proliferation markers, i.e., proliferating cell nuclear antigen and Ki-67 [16]. Inactivation of SMARCA2 and SMARCA4 in a mouse model resulted in accelerated lung tumor development and loss of differentiation, closely resembling human lung cancer characterized by local tumor invasion and distant metastasis [17].

### Association of SMARCA2 and SMARCA4 deficiency with other important cell markers in lung cancer

Loss of SMARCA2 and SMARCA4 was frequent in solid-predominant adenocarcinomas and tumors with low levels of bronchial epithelial markers, i.e., TTF-1, CK7, and E-cadherin. Interestingly, only the loss of SMARCA2 was found concomitant with the presence of tumor lepidic components and epidermal growth factor receptor (EGFR) mutations, whereas the loss of SMARCA4 was mutually exclusive with those findings [7]. However, although EGFR is usually not mutated in SMARCA4-deficient NSCLC, a case with an EGFR mutation was previously reported [18]. Loss of TTF-1 was also demonstrated in the vast majority (80%) of SMARCA2/SMARCA4-deficient adenocarcinomas in the study by Herpel et al. [10].

Significant histopathological diversity with inflammatory infiltration and tumor necrosis characterizes SMARCA4-deficient NSCLC. In a case series of 47 patients with the disease, immunohistochemistry revealed the distinct immunophenotype TTF-1-/napsin A^−^/CK7^+^ in 60.9% of the patients, positive hepatocyte paraffin 1 in 46.5%, and concomitant reduction or loss of SMARCA2 in 11.8%, whereas the loss of AT-rich interactive domain-containing protein 1A or 1B was not found in any case [19]. In another two case series of cumulatively 15 patients, immunohistochemical analysis showed positive CK7, negative p40, and negative TTF-1 in all or most cases [20, 21].

Previous studies have shown that *SMARCA4* mutations in lung cancer co-segregate with other clinically important genes. The expression of multiple genes is regulated by SMARCA4, and their transcription is repressed with the inactivation of SMARCA4 in lung cancer [22]. *SMARCA4* mutations coexist with mutations in *KRAS, LKB1, NRAS, P16,* and *P53*, as well as with the absence of *MYC* amplification [6]. Romero et al. showed that SMARCA4 regulates the expression of *MAX,* a gene that encodes the MYC-associated factor X (MAX), which is inactivated in SCLC. SMARCA4 is necessary for MAX to activate neuroendocrine transcriptional programs and to upregulate MYC targets, such as glycolysis-related genes. They also observed that experimental depletion of SMARCA4 led to decreased cell viability in MAX-deficient cells, setting the background for potential therapeutic strategies in SCLC demonstrating MAX deficiency [23]. On the other hand, Schoenfeld et al. [24] identified mutations of *SMARCA4* in 8% of 4813 cases of NSCLC and categorized them into class 1 (truncating mutations, fusions, and homozygous deletion) and class 2 (missense mutations). They further showed that SMARCA4 mutations of both classes coexisted with mutations in *KEAP1, KRAS,* and *STK11* more frequently compared to *SMARCA4* wild-type tumors, whereas class 1 mutations were associated with poor clinical outcomes compared to class 2 mutations.

### SMARCA2- and SMARCA4-related therapeutic strategies in lung cancer

Previous studies have shown promising results regarding SMARCA2/SMARCA4-related personalized therapeutic strategies in NSCLC. The loss of SMARCA2 or SMARCA4 in NSCLC resulted in reduced expression of cyclin D1. Importantly, the loss of SMARCA4 was found to be synthetic lethal with the inhibition of cyclin-dependent kinase 4/6 or aurora kinase A, highlighting their inhibitors as potential therapeutic agents in SMARCA4-deficient NSCLC cases [25, 26]. Despite the fact that the loss of SMARCA4 was associated with an unfavorable prognosis, it has been suggested as a novel prognostic biomarker for predicting outcomes in NSCLC cases on platinum-based chemotherapy after tumor resection, with improved five-year disease-specific survival noted in patients with low *SMARCA4* expression [27]. Additionally, targeting SMARCA2 in NSCLC cell lines bearing mutations in *SMARCA4* was associated with increased responsiveness to radiotherapy [28].

The use of immunotherapy in SMARCA4-deficient undifferentiated thoracic tumors is recommended as a first-line, second-line, or later treatment depending on the expression of PD-L1 [29]. On the contrary, it remains questionable whether immunotherapy has a therapeutic role in NSCLC with mutations in *SMARCA4*. A small number of studies in the literature have shown promising results regarding the use of immunotherapy. The addition of programmed cell death protein 1 (PD-1) immune checkpoint inhibitors (ICIs) to chemotherapy in patients with stage III-IV SMARCA4-deficient NSCLC resulted in better one-year survival and progression-free survival in a small study from China [30]. In another study, eight SMARCA4-deficient NSCLC patients received PD-1 or programmed death ligand 1 (PD-L1) ICI therapy, and four of them demonstrated a sustainable clinical response [19]. Furthermore, treatment with PD-1/PD-L1 ICIs in another two patients with SMARCA4-deficient NSCLC led to marked tumor regression and improved performance status [31].

On the other hand, several studies in the literature point toward a neutral or negative role of immunotherapy in NSCLC or SCLC with SMARCA4 deficiency. The efficacy of immunotherapy did not show a significant difference between *SMARCA4*-mutant and *SMARCA4*-wild-type NSCLC in a series of 19 and 280 patients, respectively [32]. This finding was also confirmed in lung adenocarcinoma through the analysis of data from 55 and 311 patients, respectively, from the cBioPortal database [33]. Importantly, concurrent mutations in *SMARCA4* and *KEAP1*, *KRAS*, or *STK11* genes were found to further enhance the resistance of NSCLC tumors to immunotherapy [32–34]. Furthermore, the administration of nivolumab in the only reported case of SMARCA4-deficient SCLC led to hyperprogressive disease, with worsening pleural effusion and chest wall dissemination of the tumor [35].

Thus, there are overall contradictory findings regarding the effect of *SMARCA4* mutations on the response to immunotherapy in NSCLC, with only a subset of studies showing better outcomes for patients. Meanwhile, only one case of SMARCA4-deficient SCLC receiving an ICI has been reported, highlighting the need for further research on the efficacy of immunotherapy in all types of lung cancer with the loss of SMARCA4.

## Conclusion

The absence of SMARCA2 or SMARCA4 in NSCLC is well-documented and correlated with poor clinical outcomes in the literature. Several studies involving clinical cases, cell lines, and animal models of lung cancer indicate a worse prognosis associated with SMARCA2 and SMARCA4 deficiency in the disease. More importantly, there are a few reports suggesting that this phenotype may predict response to treatment. We describe a rare case of SCLC with a loss of SMARCA4 in a patient with rapidly progressive disease, indicating that, as in NSCLC, patients with SMARCA4-deficient SCLC may experience aggressive disease and reduced survival compared to cases with preserved SMARCA4. This observation is clinically significant in the context of personalized medicine, as SMARCA2 and SMARCA4 in NSCLC and SCLC may be utilized in the future as biomarkers for disease progression. Their expression may assist clinicians in selecting the most appropriate therapeutic option for each individual patient.

## Data Availability

No new data were created or analyzed in this study. Data sharing is not applicable to this article.
